# Formulation of Hydrogel Beads to Improve the Bioaccessibility of Bioactive Compounds from Goldenberry and Purple Passion Fruit and Evaluation of Their Antiproliferative Effects on Human Colorectal Carcinoma Cells

**DOI:** 10.3390/gels11010010

**Published:** 2024-12-27

**Authors:** Ana María Naranjo-Durán, Diego Miedes, Juan Manuel Patiño-Osorio, Antonio Cilla, Amparo Alegría, Catalina Marín-Echeverri, Julián Quintero-Quiroz, Gelmy Luz Ciro-Gómez

**Affiliations:** 1 Group of Toxicology, Food and Therapeutic Alternatives, College of Pharmaceutical and Food Sciences, University of Antioquia UdeA, Calle 67, Medellin 053108, Colombia; juanm.patino@udea.edu.co (J.M.P.-O.); catalina.marin@udea.edu.co (C.M.-E.); jquinteroq@ces.edu.co (J.Q.-Q.); gelmy.ciro@udea.edu.co (G.L.C.-G.); 2 Nutrition and Food Science Area, Faculty of Pharmacy and Food Sciences, University of Valencia, Av. Vicente Andrés Estellés s/n, 46100 Burjassot, Valencia, Spain; diego.miedes@uv.es (D.M.); amparo.alegria@uv.es (A.A.); 3 College of Sciences and Biotechnology, CES University, Calle 10 # 22-04, Medellin 050018, Colombia

**Keywords:** bioaccessibility, bioactive compounds, goldenberry, purple passion fruit, hydrogel beads, antioxidant activity, antiproliferative activity, Caco-2 cells, polyphenols

## Abstract

Goldenberry and purple passion fruit contain bioactive compounds (BCs) that can prevent gastrointestinal cancers; hydrogel beads can protect and control their release in the gastrointestinal tract. This study aimed to develop an encapsulating material for fruit hydrogel beads (FHBs) to increase their bioaccessibility and to assess antiproliferative effects. A blend of goldenberry–purple passion fruit was encapsulated using ionic gelation and electrospraying. Through a mixture experimental design with sodium alginate (SA), hydroxypropylmethylcellulose (HPMC) and arabic gum (AG) as components, the following response variables were optimized: polyphenol bioaccessibility and encapsulation efficiency. Polyphenols and antioxidant activity were quantified before and after digestion. Antiproliferative effect was evaluated on Caco-2 colon cancer cells. Variations in formulation proportions had a significant effect (*p* < 0.05) on most responses. An SA-AG mixture in a 0.75:0.25 ratio maximized polyphenol bioaccessibility to 213.17 ± 19.57% and encapsulation efficiency to 89.46 ± 6.64%. Polyphenols and antioxidant activity were lower in FHBs than in the fruit blend (F). Both F and FHBs inhibited tumor cell proliferation by 17% and 25%, respectively. In conclusion, encapsulating BCs in hydrogel beads with SA-AG can enhance the effectiveness of polyphenols in food applications by improving their bioaccessibility and showing a more pronounced effect in inhibiting tumor cell proliferation.

## 1. Introduction

Healthy dietary patterns with a proper consumption of antioxidant-rich foods such as fruits and other plant-based foods [1] help prevent noncommunicable diseases (NCDs), which are the main cause of death worldwide [2], like cancer of the digestive tract, among others. This global challenge has sparked increasing interest in healthy eating habits and guided research toward functional food compositions aimed at developing new products containing bioactive compounds (BCs). Exotic fruits cultivated in Colombia like goldenberry (*Physalis peruviana* L.) and purple passion fruit (*Passiflora edulis f. edulis Sims*) are rich in BCs such as antioxidant vitamins (vitamin A and C), polyphenols and carotenoids [3,4]. Carotenoids belong to a group of phytochemicals with essential functions in human health, as some serve as vitamin A precursors and possess the ability to act as antioxidants, reducing reactive oxygen species (ROS) [5]. In turn, polyphenolic compounds are one of the biggest groups of BCs in foods, exerting numerous biological effects (antioxidant, anti-inflammatory and antiproliferative effects). Many studies have been carried out regarding their antiproliferative activity; however, they have commonly been carried out with isolated polyphenols, which is not realistic and may overestimate the effect [6]. More recent studies have explained the mechanisms by which they undergo metabolism and are then made bioavailable in the human body [7]. BCs’ bioavailability, bioaccessibility and bioactivity is an increasingly important area of research. Bioavailability encompasses both bioaccessibility and bioactivity, which includes processes such as digestion, metabolism, tissue distribution, absorption, biotransformation and the physiological response of the human body. However, determining the actual bioavailability of certain BCs in foods can be challenging due to practical limitations and ethical considerations in research. Consequently, much of the research in this field focuses on bioaccessibility, which refers to the portion of BCs released from foods in the gastrointestinal tract (GIT) that have the potential to be absorbed and become bioavailable. Bioaccessibility is a key factor influencing the bioactivity of BCs in various functional food. Additionally, the bioactivity of BCs measured in vitro can differ substantially from that observed in vivo. This fact can be attributed to the lower bioaccessibility due to a deficient stability of BCs in the GIT and a hard absorption through cell membranes [7]. Furthermore, the bioaccessibility of BCs is affected by the complex relationship among BCs, the food matrix and the GIT and by interaction with other nutrients or factors needed for digestion (pH, digestive enzymes, gut microbiota, bile salts, etc.) [8,9,10]. The INFOGEST method offers a static in vitro approach, featuring precisely controlled, enzymatic activities, salt concentrations and pH levels, to simulate oral, gastric and intestinal digestion [11]. Investigating the interaction between food digesta and the gut barrier in vitro is essential, especially for isolating the bioavailable fraction for assays using cell models outside the GIT, or for mechanistically assessing the fate and efficacy of BCs, among other factors [10].

Considerable efforts are being made to enhance the impact of BCs on human health and to ensure the efficacy of foods formulated with these compounds. For this reason, some mechanisms have been evaluated to increase water solubility, stability in GIT and capacity to arrive at its target site within the body and produce a beneficial effect on human health [12]. Hydrogel-formulated beads represent a solution to this challenge, since they can protect BCs with a strong and semipermeable membrane [13] and have been shown to overcome solubility incompatibility between ingredients, protect BCs against degradation and improve bioavailability [14]. Hydrogel beads can be produced through electrospray-assisted ionic gelation, an emerging technology that arose for incorporating BCs in polymeric particles with food and pharmaceutical industry applications. Unlike other techniques, ionic gelation does not require high temperatures or organic solvents that could affect the stability of sensitive nutrients or raise toxicity concerns. Other advantages include its aqueous interior, uniform distribution of BCs throughout the matrix, ease of handling and industrial scalability, making it a promising technique that also complies with green industry ecological standards [14,15,16]. Electrospray is a method of atomizing a liquid solution through an electrical field; droplets extruded break apart into smaller droplets due to internal electrostatic repulsions [16]. In the case of ionic gelation, finer particles made up of a polymeric solution fall into the gelling agent solution where the process of gelation occurs, resulting in the formation of HB. For example, Nikoo and colleagues [16] achieved a reduction in particle size of calcium alginate hydrogel beads by electrospray from 2740 ± 115 μm to 765.29 ± 14.53 μm. These HB are typically composed of sodium alginate (SA), a polymer made up of α-L-guluronic acid and β-D-mannuronic acid. Its water solubility is pH-dependent; this value determines the charge of the residues [17]. If they are negatively charged, gel will be formed in the presence of small amounts of polyvalent ions, where biopolymer chains are arranged in a parallel and symmetrical way, forming negatively charged cavities filled with cations, forming the structure known as “egg box”, i.e., alginate solubility decreases in an acidic medium. Through the use of arabic gum (AG) and other materials, it is possible to improve the efficiency of the encapsulation (EE) process [7]. For example, AG creates a dry cover around the material of the nucleus, preventing contact with air. Although AG’s structure has not yet been fully identified, it consists of D-galactopyranosyl units that form the main chain, with lateral chains branching off from it. On the other hand, hydroxypropyl methylcellulose (HPMC) can reduce the rate at which water enters the polymer matrix [18]. Additionally, fruit pectins are high-molecular-weight polysaccharides made up of long, regular chains of 1,4-linked D-galacturonate, which can interact with the polymeric chains of SA [19].

Studies on the biological activity of phytochemicals, including their antiproliferative effects, are abundant, particularly when examining isolated polyphenols. However, there is a notable scarcity of research conducted on fruit blend extract in HB as a functional ingredient subjected to in vitro gastrointestinal digestion [20]. Considering the points mentioned above, this study aimed to improve the bioaccessibility of BCs and its antioxidant activity from a blend of exotic Colombian fruits through the formulation of a polymer mixture to obtain particles by electrospray-assisted ionic gelation and to evaluate the antiproliferative activity of bioaccesible fractions (BFs) in a colorectal cancer cell line (Caco-2).

## 2. Results and Discussion

### 2.1. Load Choice

**Figure 1:**
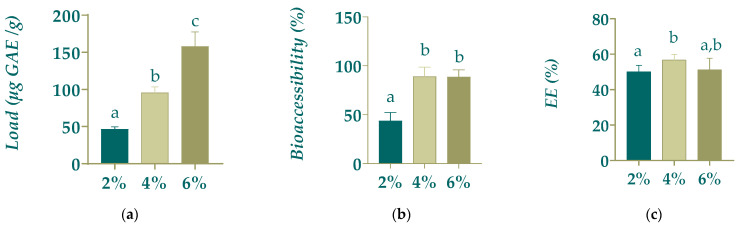
Effect of the percentage of fruit solids in the hydrogel beads formulation on (**a**) the load of the beads, (**b**) the polyphenol bioaccessibility and (**c**) the polyphenol encapsulation efficiency (EE). Different letters indicate statistically significant differences (*p* < 0.05).

Analysis of variance of the results from the evaluation of three different solids concentrations of fruits loaded in alginate beads showed that polyphenol load (*p*-value = 0.0000) as well as polyphenol bioaccessibility (*p*-value = 0.0038) exhibited statistically significant differences between the studied solids concentrations. In addition, the highest polyphenol load (162.97 ± 15.82 µg gallic acid (GA)/g _beadss_) was obtained with a 6.0% solids concentration, followed by a polyphenol load of 94.39 ± 7.34 GA mg/g _beads_ for the 4% ones and 45.46 ± 2.51 GA mg/g _beads_ for the 2% ones (Figure 1a). For polyphenol bioaccessibility, Figure 1b indicates no statistically significant differences between 4.0% and 6.0% beads (88.88 ± 7.92% and 88.61 ± 5.90%, respectively); however, there were statistical differences regarding the 2.0% beads with a much lower value (43.62 ± 6.16%). Lastly, even though polyphenols encapsulation efficiency (EE) of 2% beads was statistically different from the 4.0% beads (50.16 ± 3.29% vs. 56.74 ± 3.20%), it does not show statistical differences regarding the 6.0% beads (51.29 ± 5.72%) (Figure 1c). According to these results, the best formulation for subsequent assays with the hydrogel beads contains 6.0% fruit solids.

### 2.2. Hydrogel Particles Formulation

**Figure 2:**
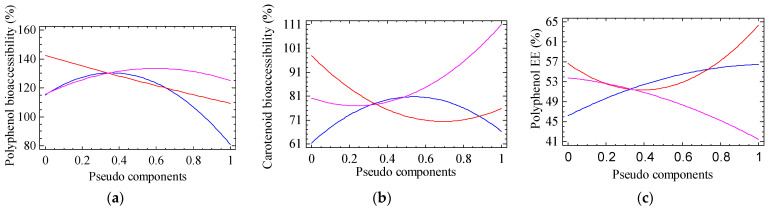

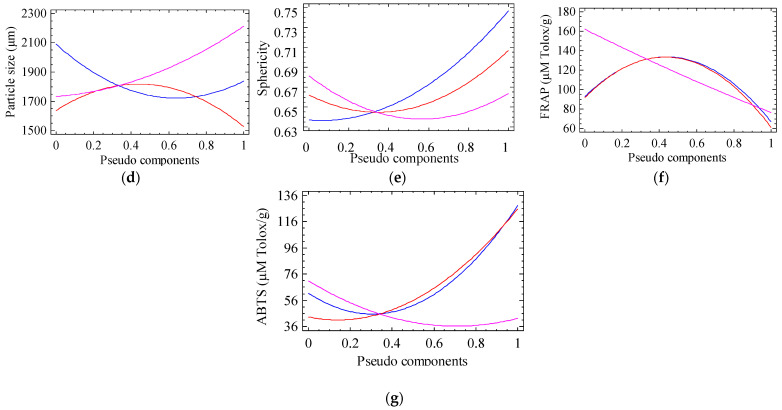
Trace graphics for (**a**) polyphenol bioaccessibility, (**b**) carotenoid bioaccessibility, (**c**) polyphenol encapsulation efficiency (EE), (**d**) Particle size, (**e**) Sphericity, (**f**) FRAP and (**g**) ABTS. Blue line represents the proportion of sodium alginate in the blend, red line the proportion of hydroxypropyl methylcellulose and pink line the proportion of Arabic gum.

Table 1 shows the results for the experimental design for every studied response. All of them exhibit an adjusted r^2^ above 70% except for polyphenol EE, and the formulation of the beads has a statistically significant effect on all of them (*p* < 0.05) except for carotenoid bioaccessibility (*p* = 0.1823). The results for polyphenol bioaccessibility, ABTS and FRAP show data above 100%. This could be explained by the transformation of the polyphenols at GIT since these compounds can be hydrolyzed because of enzyme and pH changes. Some phenolic compounds may undergo structural changes due to pH fluctuations during digestion, particularly citrus phenolic acids like ferulic acid. These compounds may elevate their concentration after digestion, as proteases (such as pepsin) and low pH (≤3) can promote the release of the phenolic acid and help maintain its chemical stability within the food matrix [21]. Additionally, these results are in concordance with those presented by Agudelo et al., 2018 [21], who reported a 2.81-fold increase in caffeic and ellagic acid bioaccessibility during simulated gastric digestion of Andean blueberry (*Vaccidium meridionale*). Similarly, some polyphenols like cyanidin-3-rutinoside increased by about 460% in wild blackberries and 314% in cultivated blackberries during gastric digestion only, and petunidin-3-glucoside in wild blackberries had a bioaccessibility of 629%. In addition, vanillic acid iso-1 had a bioaccessibility of 118% and 842% for cultivated and wild berries, respectively, while vanillic acid iso-2 had a bioaccessibility of 175% and 30% for cultivated and wild berries, respectively [22]. Digestion conditions are key factors driving the biotransformation of high-molecular-weight polyphenols. Therefore, digestion boosts the formation of phenolic metabolites (low-molecular-weight phenolics), improving their bioaccessibility, which could directly influence their bioavailability and bioactivity. Figure 2a,b present trace plots for a reference blend at intercept (0.66-0.17-0.17) for SA-HPMC-AG. Increasing the proportion of AG in the bead’s formulation favors the bioaccessibility of both polyphenols (Figure 2a) and carotenoids (Figure 2b). In the same way, it was found that by incorporating AG in a lyophilized formulation of orange juice co-products extract improves the bioaccessibility of polyphenols and carotenoids [23].

The range of the values for polyphenol EE (40.8–64.7%) (Table 1) was similar to those reported by encapsulating polyphenols from a grape pomace extract (40.08%) using SA only, and obtained an increase in EE combining SA with gelatin and chitosan to 52.63 and 56.25%, respectively [24]. However, for experimental run #1, the beads formulated with 0.5 SA and 0.5 HPMC achieved a higher polyphenol EE (64.7%). The solubility of both the coating material and the active ingredient, along with their interactions, plays a crucial role in determining the activity. The behavior of these interactions can be seen in Figure 2c, which represents trace plot for a reference blend at intercept (0.66-0.17-0.17) of SA-HPMC-AG. Both the increase in the proportion of HPMC (red line) as well as the increase in SA (blue line) favors polyphenol EE, and the increase in the proportion of AG (pink line) reduces it. This behavior could be due to the fact that HPMC can reduce the water permeability rate in the polymer matrix; thus, it reduces the loss of polyphenolic compounds by decreasing their effective diffusivity through the hydrogel microstructure [18].

The particle size (PS) and sphericity (S) of produced hydrogel beads are physical properties influenced by the encapsulation method and the coating material used, which in turn affect the pharmacokinetic and pharmacodynamic properties of the encapsulated substance. For example, higher EE beads typically have greater thickness, resulting in slower release, whereas smaller beads, with a larger surface area, may facilitate faster release of the BCs [24]. The lower PS was achieved for run #10 with formulation (0.67-0.17-0.17), and the highest S values correspond to runs 6 and 11 with 0.759 and 0.739, respectively (Table 1). The formulations of these two runs are composed only of SA. Similarly, Martinović et al., 2023 [24], found that SA microbeads exhibited the highest circularity values, a measure of particle deviation from sphericity, indicating that the SA microbeads were more spherical than both alginate–gelatin and alginate–chitosan microbeads.

The optimization of the experimental design, aimed at maximizing the bioavailability of the CBs, resulted in a desirability of 92% for the most suitable formulation, which consists of 0.75 SA and 0.25 AG. AG is a complex heteropolysaccharide with a highly branched structure and a negative charge at pH values above 2.2. It consists of three main fractions: glycoprotein, arabinogalactan-protein and arabinogalactan-peptide, which contribute approximately 2%, 10% and 85–90% of the total mass, respectively. Of these fractions, less than 1%, around 10% and 25–50% are composed of protein, respectively. Therefore, the majority of the AG molecule is composed of polysaccharides, with the protein content varying significantly across the different fractions. On the other hand, alginate is negatively charged at a pH above 3.5. Although there is no strong electrostatic attraction between these two negatively charged biopolymers, AG can interact with alginate at pH 4. In this case, the interaction between SA and AG fractions is primarily driven by electrostatic forces, where positively charged regions in AG protein (or its fractions) interact with the negatively charged carboxylate groups of SA [25]. The possibility of hydrophobic interactions and hydrogen bonding cannot be ruled out at this stage. This formulation (0.75 SA and 0.25 AG) managed to maximize polyphenol bioaccessibility to 213.17 ± 19.57%; polyphenol EE to 89.46 ± 6.64%; the antioxidant activity recovery by ABTS to 68.38 ± 0.00% and by FRAP to 95.62 ± 11.81%; and the S to 0.69. At the same time, it decreased the PS to 1194.48 ± 167.34 µm. The relative errors of the response variable are between 26.77 and 37.10%, except for FRAP and S, which had a relative error of approx. 4%. In this sense, spray-drying encapsulation of chokeberry polyphenols using AG as carrier obtained a polyphenol EE of 85.29 ± 6.15%, which is in accordance with our results [26].

**Figure 3:**
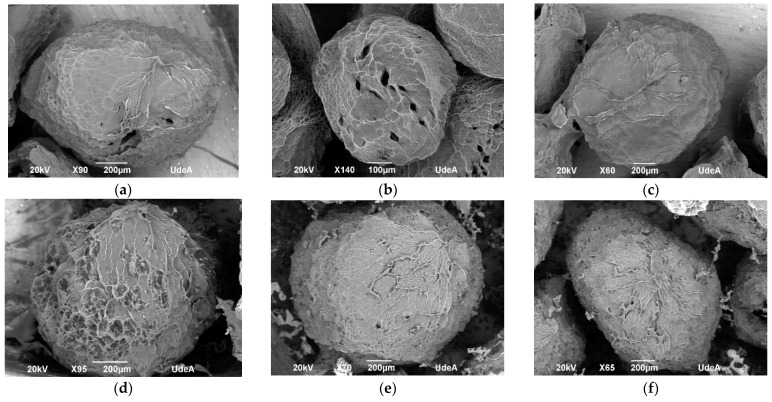
SEM micrographs (**a**–**c**) for hydrogel beads and (**d**–**f**) for fruit blend hydrogel beads.

SEM micrographs (Figure 3a–c) show uniform, spherical and semispherical morphology for the hydrogel beads (HB), and Figure 3d–f for the fruit blend hydrogel beads (FHBs). The smooth surface of the HB is the most different characteristic among them, with a porous structure with cavities. The incorporation of a fruit blend (F) into the hydrogel beads resulted in a rougher surface and fewer cavities inside compared to those without the fruit blend, as shown in Figure 3b,e. SEM micrographs also showed a PS according to the obtained one by ImageJ 1.45s ^®^software (1194.48 ± 167.34 µm).

### 2.3. Antioxidant Activity of the Hydrogel Beads Loaded with Fruit Blend

The antioxidant activity of the F, FHBs and HBs are shown in Table 2. Total polyphenolic content and the antioxidant activity in non-digested samples measured by ABTS, FRAP and ORAC in the FHBs are lower than those of F, with statistically significant differences. This fact could be because the process of FHB production has a polyphenol EE of 89.46% and that the release of the BCs in the GIT was probably not complete. In general, the bioactivity of fruits is directly related to their chemical profile; in this study, 35 polyphenols were identified in total for both fruits shown in Table 3. In this sense, catechin, epicatechin and rosmarinic acid are the ones with highest concentration in a methanolic extract of Colombian purple passion fruit [27], and rutin and chlorogenic acid in goldenberry [28].

The polyphenolic bioaccessibility of the F increased after the digestion process by 1.13-fold, while the FHBs increased by 2.22-fold; however, this significant increase may be due in part to the polyphenolic content presented on the HBs after the digestion process, as a result of the fact that the method used to quantify polyphenols does not have a high specificity, and after in vitro gastrointestinal digestion, there may be interferences. This result is in accordance with a 1.26-fold increase in total polyphenolic content from *Rubus hirsutus Thunb* at the end of GIT [29]. Similar behavior was found for the antioxidant activity measured by ORAC. The value of ORAC for the F was 47.06 ± 9.31 μM Trolox/100 g and its BF was 7865.90 ± 1238.55 μM Trolox/100 g (Table 2), which represents a 167-fold increase. In the case of FHBs, the increase was of 479-fold, and for the HBs, the increase was 1006-fold. Moreover, in the previous study conducted by our research group [30], for freeze-dried extracts in fresh weight, the ORAC value for goldenberry was 2302.0 ± 36 μM Trolox/100 g and for purple passion fruit was 3244.0 ± 94 μM Trolox/100 g. According to these reports, the ORAC value for goldenberry-purple passion fruit blend (0.83:0.17 ratio) should be 2859.57 μM Trolox/100 g dry weigh, but in this case, it was 47.06 ± 9.31 μM Trolox/100 g in fresh weight or 784.33 μM Trolox/100 g in dry weight. The theorical value is 3.65-fold greater that the experimental one, and this may indicate an underestimated value. The behavior above may be due to possible interactions like the formation of hydrogen bonds between SA and polyphenol molecules [31] or the interaction of the hydroxyl or carboxylate groups from the SA with different polar groups of the polyphenols [32,33]. On the other hand, polyphenols can bind non-covalently to polysaccharides like AG, affecting the binding capacity, molecular weight, structural flexibility and number of OH that can affect the antioxidant measure. Besides BCs, fruit extracts include non-starch polysaccharides such as cellulose or hemicellulose, lignin and pectin, which could also contribute to this interaction [26,33]. Thus, the ORAC methodology is also used as a tool to simulate the antioxidant activity of phenols in biological systems; all these interactions can explain the great difference between the ORAC values of the samples and their BFs (Table 2).

**Figure 4:**
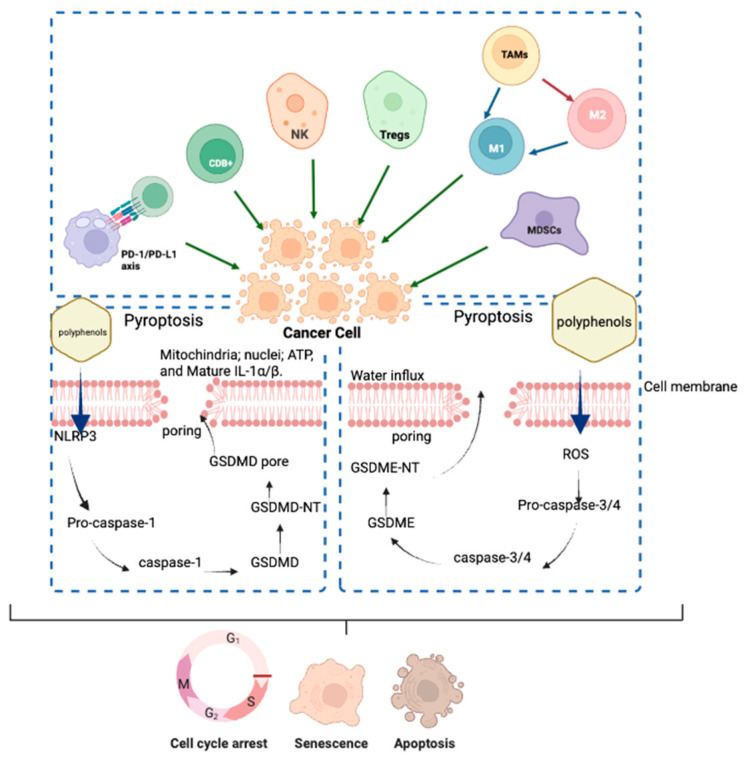
Action mechanism of polyphenols on inhibiting tumor cell proliferation.

Some of these polyphenols, including apigenin, demonstrated anticancer activity by inducing apoptosis and arresting the cell cycle [34], and in the same way, catechin [35] rutin [36], rosmarinic acid [37], caffeic acid and chlorogenic acid [38] have demonstrated anticancer activity in different cell lines. The anticancer activity of these polyphenols is related to its antiproliferative, antioxidant and anti-inflammatory activity, i.e., phenolic rings can accept electrons to form stable phenoxyl radicals, which protect cells from oxidative damage. Moreover, polyphenols exhibit dual mechanisms for inducing cancer cell death through pyroptosis pathways. The first mechanism involves the activation of the NLRP3 inflammasome, which triggers pro-caspase-1 activation, leading to GSDMD cleavage and subsequent pore formation. The second pathway operates through ROS generation, activating pro-caspase-3/4, which cleaves GSDME to form membrane pores. These mechanisms work in concert with immune system components, including NK cells, CD8+ T cells and macrophages (M1/M2), while being regulated by TAMs and affected by the PD-1/PD-L1 axis. The ultimate outcomes include cell cycle arrest, senescence and apoptosis, demonstrating the comprehensive anti-cancer effects of polyphenolic compounds (Figure 4) [39].

Phenolic compounds are stored in the cell structures of fruits, where they are bound to macromolecule-like proteins through the phenolic group to the –NH group of peptides, or carbohydrates by α- and β-glycosidic bonds. These macromolecules undergo significant degradation in the GIT, breaking down into small peptides or amino acids, bioavailable sugars and phenolic compounds, which often leads to the release of the most bioaccessible forms of BCs [40]. For instance, vanillic acid isomers can be produced through the spontaneous cleavage of anthocyanidins (like cyanidin present in goldenberry and purple passion fruit extracts (Table 3)). Likewise, ferulic acid is an intermediate product of the catabolism of flavonoids, while caffeic acid is a hydroxycinnamic acid, with its main metabolites being vanillic acid isomers, which can be formed through the spontaneous cleavage of protocatechuic acid [22]. All these reactions may explain the increase in polyphenols after gastrointestinal digestion. In general, the abovementioned behavior may be due to the fact that after the gastrointestinal digestion process of these beads, antioxidant compounds and other BCs can be transformed into different forms with varying bioaccessibility and biological activity, which may impact the original antioxidant potential of each component [20], including the biopolymers used for the fabrication of the hydrogel beads. For instance [33], ORAC values for different samples of AG have been reported at 1877 ± 7 µM Trolox/100 mg, 976.6 ± 4.8 µM Trolox/100 mg and 3266 ± 7 µM Trolox/100 mg, and the compounds that exert this antioxidant activity can also be transformed into different forms after gastrointestinal digestion with higher antioxidant activity. Additionally, various studies have found that AG could enhance the antioxidant status of the human body by protecting the liver through the modulation of oxidative stress-related gene expression. These results suggest that AG and alginate may have relevant antioxidant activity after the digestion process.

### 2.4. Antiproliferative Effect on Human Colorectal Carcinoma Cells

**Figure 5:**
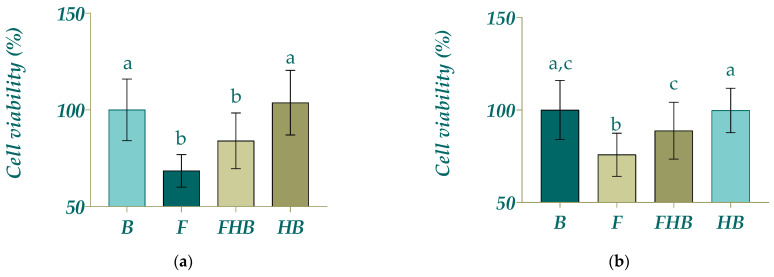
Effect of bioaccessible fractions of samples (diluted 1:15 *v*/*v* with DMEM) (fruit blend (F), fruit hydrogel beads (FHBs) and hydrogel bead in blank (HB)) on the cell viability of Caco-2 cell lines, (**a**) non-differentiated as a tumoral model and (**b**) differentiated as an intestinal epithelial-like non tumoral model. Different letters indicate statistically significant differences (*p* < 0.05).

Given the positive correlation that is suggested between the phenolic content, antioxidant capacity and the antiproliferative activity [41], the BFs of HBs, FHBs, F and blank of digestion (B) were screened for their antiproliferative potential in colon cell lines (non-differentiated Caco-2 as a tumoral model and differentiated Caco-2 cells as an intestinal epithelial-like non-tumoral cell model) (Figure 5). Considering the fact that a small decrease in cell viability is possible when including the B, possibly due to the remaining enzymes in the digests [41], the values of cell viability obtained with the B were used as reference values instead of the control. This comparison provides more accurate results regarding the actual activity of the antioxidants present in the BFs of the samples. The FHBs and F were able to inhibit the proliferation of the tumoral cell model (17 and 25%, respectively) compared with the control (untreated cells) (*p* < 0.05) without statistically significant differences between them, while HBs and B did not differ from control cells (*p* > 0.05) (Figure 5a). On the other hand, for the non-tumoral cell model, only F had a slight significant cytotoxic effect on cell viability compared to control cells (*p* < 0.05) (Figure 5b), but this was less pronounced than in the tumoral cell model, indicating a selective effect of samples in the tumoral Caco-2 cell model. In the same way, aqueous extracts from yellow passion fruit on the SW480 cell line from human colon adenocarcinoma shown a cytotoxic activity dose-dependent response; the treatment with 10% extract (264 µg/mL) showed the greatest reduction in cell viability, with a decrease of 16.98% [39].

The results shown in Figure 5 indicate that the highest antiproliferative effects were obtained with the F and FHBs, which may demonstrate that the encapsulation of BCs does not diminish their antiproliferative activity, despite the prior results obtained for the antioxidant activity and for the polyphenol content, where a lower polyphenol content and antioxidant activity were found for FHBs than for F. Additionally, the slight non-significant lower antiproliferative effect of FHBs vs. F could be attributed to the fact that the BCs had not been completely released from the bead into the medium; furthermore, it may be that the dose released had not reached the effective dose to reduce cell viability as much as in the F sample. These results suggest that F and FHBs may have a co-adjuvant effect for the treatment of colon cancer as reported by Hassan et al., 2017 [3], where goldenberry juice showed chemosensitizing activity for the treatment with adriamycin of hepatocellular carcinoma induced in a murine model.

Allowing that, Zayed et al., 2022 [34], found an antiproliferative effect of the apigenin in a concentration-dependent manner in HePG-2 (hepatocellular carcinoma) and Hela cells, and in the same way, Tsai and Chen, 2016 [42], report an antiproliferative effect with dose-dependent decrease for the for the cell viability in PC-3 (prostate adenocarcinoma cells) and CCD-986SK (fibroblast cells) after treatment with free catechin extract or catechin in nano-emulsion. A high cell viability of 92.7% was observed for the free catechin dose at 2.5 µg/mL. However, as the catechin extract dose was increased to 5 µg/mL, 7.5 µg/mL, 10 µg/mL, 20 µg/mL and 30 µg/mL, cell viability decreased to 92.1%, 75.7%, 75.2%, 28.6% and 5.9%, respectively, for PC-3 cells, and declined to 88.1%, 61.6%, 44%, 15% and 2.7% for CCD-986SK.

#### 2.4.1. Cell Death

**Figure 6:**
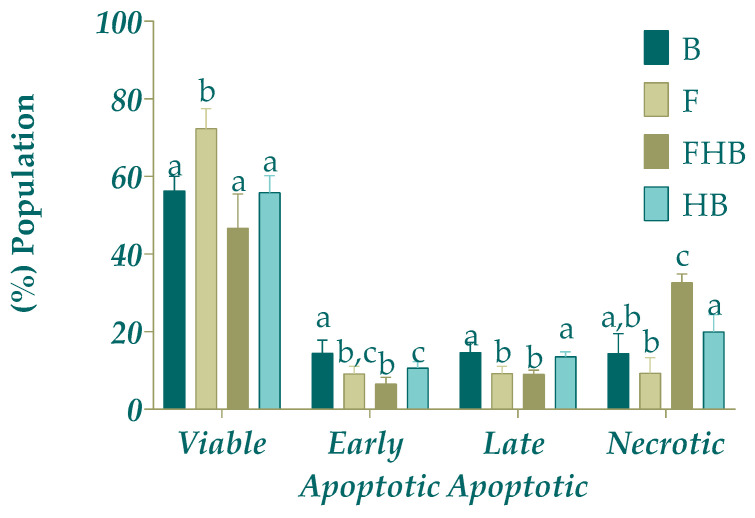
Effect of bioaccessible fractions of samples (diluted 1:15 *v*/*v* with DMEM) (fruit blend (F), fruit hydrogel beads (FHBs) and hydrogel bead in blank (HB)) on cell death status distribution of Caco-2 tumoral model line. Different letters indicate statistically significant differences (*p* < 0.05) among samples in the same cell state.

The cell death status distribution after treatment with the BFs of F, FHBs and HBs is shown in Figure 6. The percentage of early apoptotic cells for the three treatments was lower (*p* < 0.05) compared with B (digestion control). Similarly, after the treatment with F and with FHBs, the late apoptotic cells were lower (*p* < 0.05) compared with treatment B (digestion control). Nevertheless, the most relevant result was that the treatment with FHBs significantly increased (18%) (*p* < 0.05) cells in a necrotic state compared to the control and the other samples. This observation can support the antiproliferative effect observed in the MTT experiment for the FHBs. The lack of effect of F sample may suggest an antiproliferative effect linked to a cytostatic action (see Section 2.4.2) without an overtly cytotoxic impact on cells. Similarly, goldenberry calyx extracts on HT-29 cell lines (human colon adenocarcinoma) increased the percentage of cells in early apoptosis after 24 h of treatment. After 72 h, there was a notable accumulation of cells in late apoptosis (50%) and in necrosis (15%) [43] In this sense, it has been reported that in PC-3 prostate cancer cells, catechin nanoemulsion treatment induces a dose-dependent increase in both early and late apoptosis, reaching 18.7% and 7.9% at 10 μg/mL, respectively. In contrast, for the catechin extract treatment, no significant difference was observed in early apoptosis across the various doses, although a higher proportion (13%) of late apoptosis cells was found at 20 μg/mL (*p* < 0.05). Regarding necrosis, no significant differences were observed for the catechin nanoemulsion treatment across doses, while a higher proportion (5.5%) of necrotic cells was detected with the catechin extract treatment at 20 μg/mL (*p* < 0.05). The authors concluded that both catechin extract and nanoemulsion treatments led to a higher proportion of PC-3 cells undergoing early apoptosis, rather than late apoptosis or necrosis, with dose being a critical factor influencing the occurrence of apoptosis or necrosis [42].

#### 2.4.2. Cell Distribution in the Different Phases of the Cell Cycle

**Figure 7:**
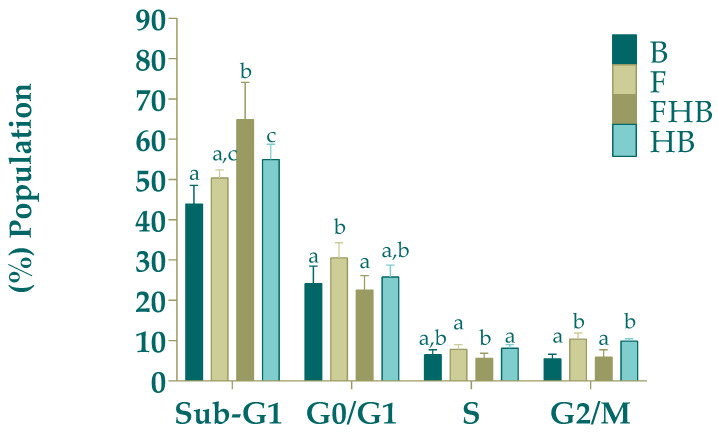
Effect of bioaccessible fractions of samples (diluted 1:15 *v*/*v* with DMEM) (fruit blend (F), fruit hydrogel beads (FHBs) and hydrogel bead in blank (HB)) on the different phases of the cell cycle of Caco-2 cell tumoral model line. Different letters indicate statistically significant differences (*p* < 0.05) in the same cell cycle phase.

The distribution of cells across the different phases of the cell cycle is represented in Figure 7. The FHBs resulted in a statistically significant increase in the number of cells in the Sub G1 phase (marker of apoptosis/necrosis) (vs. B (*p* < 0.05)), in line with the results of cell death status previously presented. Consequently, the proportion of cells in all following phases was significantly decreased, but none showed a significant difference vs. B. Regarding the F sample, an arrest was shown in G0/G1 and G2/M cell cycle phases compared to B, a finding that may be associated with a decrease in cell proliferation in line with the cell viability results in the MTT assay. The fact that it was not accompanied by an increase in subG1 may suggest a cytostatic nontoxic antiproliferative effect. Although the HB sample slightly modified subG1 and G2/M phases vs. B, these modifications were not implied in any antiproliferative activity nor apoptosis/necrosis induction.

The treatment of HT-29 cell lines (human colon adenocarcinoma) with goldenberry calyx extracts for 24 h did not result in any significant changes in cell-cycle phase distribution. However, at 48 h, there was a statistically significant increase (*p* < 0.05) in the percentage of cells in the S phase, rising to 48% compared to the control group, which showed 16.5% [43].

These results that show an arrest in some of the phases of the cell cycle associated with antiproliferative activity but without the induction of apoptosis are consistent with those reported by by Cilla et al., 2010 [6], who evaluated the antiproliferative effects of bioaccessible fractions of fruit drinks made from concentrated juices of orange, grape and apricot supplemented with iron, zinc and milk against Caco-2 cell lines. The digest of the fruit drinks supplemented with zinc and milk presented the highest anti-proliferative activity, with 35% inhibition of proliferation, and could potentially be modulated by inhibiting progression of the cell cycle in the S phase in Caco-2 cells. Additionally, Cilla et al., 2009 [20], reported an antiproliferative effect with 53% inhibition of proliferation for continuous treatment (24 h) with 7.5% of fruit beverage digest treatment, showing a halt in S phase coupled with a decrease in G0/G1. Similarly, HepG-2 cells treated with encapsulated apigenin were arrested in the S phase and exhibited a higher proportion of cells in the sub-G1 phase compared to untreated cells [34], while free apigenin induced an arrest in the G2/M phase of the cell cycle. The authors suggest that encapsulated apigenin enters the cell nucleus and binds to the DNA double strand, preventing its synthesis and leading to cell accumulation in the S phase. So, the treatment with encapsulated apigenin was associated with apoptosis, and the encapsulation altered its impact on the cell cycle. In addition, the stimulation of the p53 gene ended with caspase 9 overexpression in the HepG-2 cells treated with encapsulated apigenin compared with those treated with free apigenin in a concentration-dependent manner [34]. Other authors reported a concentration-dependent behavior for free and nano-emulsion catechin [42]. Their work showed a dose-dependent decrease in the G0/G1 phase, with values of 61.2%, 55.8% and 43.4% observed at doses of 5, 7.5 and 10 μg/mL, respectively. Notably, catechin nano-emulsion resulted in a lower proportion of cells in the G0/G1 phase compared to catechin extract at the same doses. Similarly, the S phase population also increased in a dose-dependent manner for both free and nano-emulsified catechin. Additionally, at equivalent doses, a greater proportion of cells in the S phase were found with nano-emulsified catechin than with free catechin.

#### 2.4.3. Levels of Reactive Oxygen Species (ROS) and Intracellular Reduced Glutathione (GSH)

**Figure 8:**
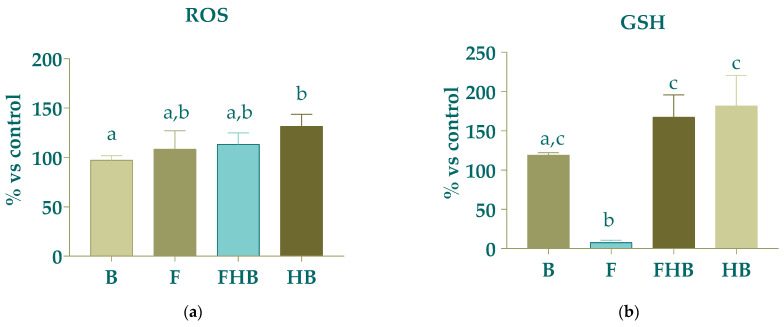
Effect of bioaccessible fractions of samples (diluted 1:15 *v*/*v* with DMEM) (fruit blend (F), fruit hydrogel beads (FHBs) and hydrogel bead in blank (HB) on (**a**) the intracellular levels of ROS and (**b**) the intracellular levels of GSH of Caco-2 cell tumoral model line. Different letters indicate statistically significant differences (*p* < 0.05).

Figure 8a displays the results for the levels of ROS. There was a tendency of elevated production of ROS without statistically significant differences for B compared to that expected for HBs. ROS levels are crucial in cancer cell metabolism, as an excess of ROS can promote cancer progression by causing DNA damage and reprogramming cellular metabolism [34]. On the other hand, the F sample evoked a significant decrease (*p* < 0.05), of over 90%, in GSH levels compared with B, suggesting an alteration in the redox status of the cells. Nevertheless, FHBs and HBs had a tendency to increase without statically significance differences in GSH compared to B. The reductions in GSH levels exerted by F might correlate with the antiproliferative effect by diminishing cellular antioxidant levels, since mitochondria dysfunction might be linked to elevated ROS levels and reduced GSH content. Recent studies have shown that certain bioactive compounds like polyphenols may reduce intracellular GSH levels and inhibit the expression of antioxidant enzymes, thereby hindering the antioxidant defenses of tumor cells. However, these compounds can also display both antioxidant and pro-oxidant effects, subject to factors such as treatment concentration, exposure duration, environmental conditions and even cell type [44].

The intracellular ROS content in Caco-2 cells after 24 h treatment with a bioaccessible fraction of broccoli and mustard microgreens showed significant increases of 94% and 62%, respectively, compared to the digestion blank; in the same way, this reduced the GSH content relative to the digestion blank, with statistical significance [45]. Similarly, treating Caco-2 cells with the BFs of fruit pulps from freshly harvested and postharvest-stored Clementine mandarins, as well as Navel and Cara cara oranges, stored at 12 °C did not result in significant increases in ROS vs. control cells. However, following pretreatment with BFs derived from citrus fruit pulps and subsequent exposure to oxidative stress with H_2_O_2_, ROS levels significantly decreased (*p* < 0.05) for mandarins, as well as for Cara cara oranges, compared to stressed control cells [46].

## 3. Conclusions

An optimal polymer blend was achieved for HB formulation, comprising 0.75 SA and 0.25 AG. This blend allowed increased polyphenol bioaccessibility and antioxidant activity recovery by ABTS and FRAP. Simultaneously, it reduced PS and increased the S. The fruit blend of goldenberry and purple passion fruit demonstrated antiproliferative potential on carcinoma cells associated with a cytostatic effect with alteration in redox status (decrease in GSH) unaffected by FHB production, with a similar antiproliferative effect ascribed to necrotic action, even though, for the non-tumoral cell model, F also had a slight cytotoxic effect on cell viability, but less pronounced than in the tumoral cell model, indicating a selective effect of samples in the tumoral Caco-2 cell model. All these results suggest that F and FHBs may have a co-adjuvant effect for the pharmacological treatment of colon cancer. However, further studies understanding the release behavior of BCs from HBs is essential to maximize their biological activity.

## 4. Materials and Methods

### 4.1. Materials

Goldenberries and purple passion fruits were purchased in the food market of Rionegro and La Ceja, Antioquia. Category two fruits were selected according to the Colombian technical standards for fresh fruits: NTC 4580 [47] for goldenberry and NTC 6456 [48] for purple passion fruit. SA, NaCl, KCl, HCl, KH_2_PO_4_, NaHCO_3_, MgCl_2_(H_2_O)_6_, (NH_4_)_2_CO_3_, CaCl_2_(H_2_O)_2_, Folin–Ciocalteu reagent, gallic acid, α-amylase from porcine, bile extract (USA), pepsin from porcine pancreas (Denmark) and pancreatin from porcine pancreas FeCL_2_, TPTZ, sodium acetate, sodium carbonate (Na_2_CO_3_), acetic acid, formic acid, β-carotene secondary standard, methanol HPLC-grade, tetrahydrofuran HPLC-grade, acetonitrile HPLC-grade and Trolox were purchased are from Sigma Aldrich, USA. Dichlorofluorescein diacetate (DCFDA), 2,2-Azobis(2-methylpropionamidine) dihydrochloride (AAPH), 3, (4,5-dimethylthiazol-2-yl)-2,5-diphenyl-tetrazolium bromide (MTT), Fluo 3-AM, fluorescein sodium salt, 5-fluorouracile (5-FU), propidium iodide (PI), ribonuclease A (RNase A) and dimethylsulfoxide (DMSO) were purchased from Merck LifeScience S.L.U. (Madrid, Spain). 5-Chloromethylfluorescein diacetate (Green CMFDA) was acquired from Abcam (Cambridge, UK). D-MEM + GlutaMAXTM (4.5 g/L glucose), MEM non-essential amino acids (MEM NEAA) solution (100×), HEPES buffer solution (1 M), antibiotic solution (10,000 U/mL penicillin and 10,000 µg/mL streptomycin), antimycotic solution (250 µg/mL amphotericin B), fetal bovine serum (FBS), PBS pH 7.4 (1×) and trypsin-EDTA solution (2.5 g/L trypsin and 0.2 g/L EDTA) were acquired from Gibco™ (Scotland, UK) FITC Annexin V Apoptosis Detection Kit I was purchased from BD Biosciencies (San Jose, CA, USA). Deionized water was obtained using a Milli-Q water purification system (Millipore™, Bedford, MA, USA).

### 4.2. Obtaining the Fruit Blend

In a cold-press extractor at 60 rpm and room temperature, the blanched fruits were processed. For a second extraction, water at 60 °C was added in a 1:5 ratio to the residues of the purple passion fruits. In the case of the goldenberries, water at 60 °C was added in a 1:5 ratio to the residues of the goldenberries and subjected to ultrasound-assisted extraction. In both cases, the two extracted phases were then combined. The mixture of these fruits, composed of 83% goldenberry and 17% purple passion fruit, was used in this study, according to the methodology described by Naranjo-Durán et al., 2023 [30].

### 4.3. Polyphenol Profile by HPLC-MS

Two grams of freeze-dried extract of each fruit was mixed with 15 mL of ethanol at 70% (*v*/*v*), which was heated at 35 °C and subjected to ultrasonic bath for 30 min; then, it was centrifuged at 5000× *g*. Finally, the supernatant was evaporated and resuspended in methanol, in a final concentration of 50 mg/mL. The resuspended extracts were filtered through a 0.22 μm pore, and 2 μL of each was injected in a HPLC- MS UltiMate 3000 (ThermoScinetific, Germering, Germany). A SiliaChrom C18 column (5 μm, 150 × 4.6 mm) (SiliaChrom, Quebec City (Quebec) Canada) was used in two mobile phases, aqueous formic acid (0.1% *v*/*v*) (A) and acetonitrile (B), with a flow rate of 0.2 mL/min, under gradient elution at 40 °C. The gradient profile was as follows: 0–1 min, 15% B; 1–7 min, 25% B; 7–9 min, 25% B; 9–13 min, 30% B; 13–16, 30% B; 16–21 min, 40% B; 21–23, 40% B; 23–25, 45% B; 25–28, 50% B; 28–33, 60% B; 33–37, 75% B; 37–38, 15% B. The identification of polyphenols was possible by comparison with reference substances and mass spectra [49].

### 4.4. Hydrogel Beads Obtention

An electrospray prototype built with a high voltage source (0–30,000 V), a stirring plate and a peristaltic pump (Velp SP 311/2, Usmate Velate, Italy) was used. The high voltage source was connected to two poles: a 12-gauge needle, fed by the encapsulating solution (SA/HPM/AG and F) through a peristaltic pump and a gelling solution of calcium chloride (CaCl_2_) at 0.03% [50]. In the distance between these two poles is produced an electric field that breaks the droplets into smaller ones, so that in contact with the gelling solution, the formation of the calcium alginate HBs begins. The following parameters were used: a height of 11.59 cm, a voltage of 12.56 kV and a flow rate of 4.33 mL/min.

### 4.5. Hydrogel Beads Formulation

A Simplex lattice mixture design was applied using STATGRAPGICS 19^®^, (The Plains, Virginia, USA), with three components (SA (0.5–1), HPMC (0–0.5) and AG (0–0.5)) and six response variables: the bioaccessibility of polyphenols and carotenoids, polyphenol EE, antioxidant activity that can be maintained in the bioaccessible fraction, PS and S, with the aim to optimize proportions in the mixes. Twelve mixtures were formulated with different proportions, as described in Table 4. To avoid SA precipitation due to a pKa = 3.65, the process was carried out at a pH of 4.5.

#### 4.5.1. Encapsulation Efficiency and Load

A total of 300 mg of beads were dissolved in 2 mL of sodium citrate 2% (*w*/*v*), and the content of polyphenols was quantified as described above. *EE* was calculated as a percentage of encapsulated *BC*s with respect to the amount of *BC*s used to obtain the beads [51]. *EE* and load were calculated with Equations (1) and (2), respectively.

(1) $$\left(\%\right)EE=\frac{ma-mb\;}{ma}\times 100$$

where *m* is the initial quantity of *BC*s, and *mb* is the non-encapsulated quantity of *BC*s.

(2) $$Load=\frac{mg\;of\;BC\;in\;hydrogel\;beads}{g\;of\;hydrogel\;beads}\times 100$$

#### 4.5.2. Morphology and Particle Size and Shape Descriptors

The beads were fixed to aluminum sample holders, and the temperature was dropped to 10 °C (Desk IV, Denton Vacuum, Moorestown, NJ, USA) [50] to determine the morphology in a scanning electron microscope (SEM, Joel 6490LV, Peabody, MA, USA) at an accelerated voltage of 20 kV. Shape descriptors were obtained by digital image analysis using a stereomicroscope and a digital camera. Approximately 12 beads were randomly selected in each picture, and their projected area and Feret diameter were calculated using the license-free software ImageJ 1.45s ^®^ USA. *PS* and *S* were analyzed according to the methodology reported by Londoño and Rojas, 2017 [52], and were calculated with Equations (3) and (4), respectively.

(3) $$PS=2\times \sqrt{area}\times \pi ,\;$$

(4) $$S=\frac{4\times area}{\pi \times {feret}^{2\;}},\;\;$$

### 4.6. Bioaccessibility of BCs from Fruit Blend in Hydrogel Beads

The 12 experimental runs were subjected to in vitro gastrointestinal digestion, and B was run in parallel, as is described below. Calcium alginate hydrogel beads were prepared with the F at 6% solids (*w*/*v*), with the optimized parameters obtained in the previous experiment. The in vitro simulated gastrointestinal release was carried out according to the INFOGEST methodology [53]: 5 g of hydrogel beads, 4 mL of simulated salivary fluid (SSF), 0.5 mL of α-amylase solution (75 U/mL), 25 µL of CaCl_2_ (0.3 M) and ultrapure water to achieve a final volume of 10 mL. The mixture was incubated at 95 rpm for 2 min at 37 °C. After that, 8 mL of simulated gastric fluid (SGF), 5 µL of CaCl_2_ (0.3 M), 0.5 mL of pepsin solution (2000 U/mL), HCl to pH 3 and water were added to make up 20 mL. This phase was left in incubation at 95 rpm for 2 h at 37 °C; finally, 8.5 mL of simulated intestinal fluid (SIF), 40 µL of CaCl_2_ (0.3 M), NaOH to pH 7, 5 mL of pancreatin solution (100 U/mL) and 2.5 mL of bovine bile salts solution were added, and ultrapure water was used to achieve a final volume of 40 mL. This mixture was left in incubation at 95 rpm for 2 h at 37 °C. Then, the samples were centrifuged at 3100× *g* for 90 min at 4 °C to separate the BF and were immediately frozen (−20 °C) to break enzymatic activity prior to analysis. The bioaccessibility of the BCs was calculated with Equation (5) [54].

(5) $$\left(\%\right)Bioaccessibility=\frac{mg\;BC\;in\;bioaccesible\;fraction\;}{mg\;BC\;in\;hydrogel\;beads\;}\times 100$$

### 4.7. Total Polyphenol Content

Total polyphenolic content was quantified by spectrophotometry following the methodology of Nunes et al. (2018) [55] with minor modifications. Briefly, 30 μL of the sample, 150 μL of 0.2 N Folin–Ciocalteu reagent and 120 μL of 7.5% (*m*/*v*) Na_2_CO_3_ were mixed and incubated at 45 °C for 15 min, followed by 30 additional minutes at room temperature in the dark. After that time, absorbance was measured at 765 nm in a UV–Vis Multiskan spectrometer (ThermoScientific, Germering, Germany). The content of total polyphenols was calculated by means of a standard curve of GA 0–110 µg/mL. All measurements were performed in fivefold.

### 4.8. Analysis of Total Carotenoid Content by HPLC-DAD

Approx. 15–20 mg of every fruit extract was dissolved in 1 mL of methanol/tetrahydrofuran (50:50 (*v*/*v*)), filtered using Nylon filters (0.45 μm) and packaged in chromatographic vials. The detection and quantification of β-carotene as the main carotenoid of the samples [30] was carried out in an UltiMate 3000 HPLC/UHPLC (ThermoFisher, Germering, Germany) chromatograph. For separation, an Accucore TM C30 2.6 μm, 150 × 2.1 mm column (ThermoFisher, Vilnius, Lithuania) was used at a temperature of 20.0 °C. A gradient elution was used with two mobiles phases, composed of methanol, tetrahydrofuran HPLC-grade and ultrapure water mobile phase A (85/5/10, *v*/*v*/*v*) and mobile phase B (11/85/4, *v*/*v*/*v*) with a flow rate of 0.5 mL/min. The gradient profile was as follows: 0 min, 2% B; 10 min, 35% B; 31 min, 73% B; 32 min, 100% B; 35 min, 100% B; 36 min, 2% B; 40 min, 2% B. The injection volume was 10 μL, and a diode array detector (DAD) at a wavelength of 454 nm was used for detection. For β-carotene quantification, a 990 ppm stock solution was prepared using methanol/tetrahydrofuran (50:50 (*v*/*v*)) as a solvent. Subsequently, dilutions (247.5; 123.8; 61.9; 30.9; 15.5; 7.70; and 3.90 ppm) were made for the calibration curve. All measurements were carried out for every run from the experimental design and for its bioaccessible fraction.

### 4.9. Antioxidant Activity

Numerous reactions contribute to the inhibition of oxidation by antioxidant compounds, including binding metal ions, scavenging ROS and deactivating singlet oxygen. Consequently, a wide array of tests exist for directly measuring hydrogen atom transfer (HAT) or single-electron transfer (SET) from antioxidants to free radicals or other oxidants. Therefore, there is no universal standard method for determining antioxidant activity. Instead, it is recommended to utilize various methods with different mechanisms, such as SET or HAT assays, to comprehensively evaluate antioxidant activity [56,57].

#### 4.9.1. Ferric Reducing Antioxidant Power (FRAP)

To determine antioxidant activity by FRAP, 35 μL of the sample and 265 μL of FRAP reagent (composed of 0.3 M buffer, 10 mM TPTZ and 20 mM Ferric chloride in a 10:1:1 ratio) were mixed in 96-well microplates and incubated in the dark at 37 °C for 30 min. Absorbance was measured at 595 nm using a Multiskan UV–Vis spectrometer (ThermoScientific, Germering, Germany). Antioxidant activity was calculated based on a standard Trolox curve (0–500 μM, r² = 1) [55]. All measurements were performed in fivefold.

#### 4.9.2. ABTS+ Anion Radical Scavenging Activity

To determine antioxidant activity by ABTS+, 10 μL of sample and 190 μL of ABTS+ radical (ABTS 14 mM–potassium persulfate 4.8 mM (1:1) in distilled water at absorbance 0.7 ± 0.02 at 740 nm) were mixed in 96-well microplates and incubated in the dark at room temperature for 6 min. Absorbance was measured in a Multiskan UV–Vis spectrometer (ThermoScientific, Germering, Germany) at 740 nm. Antioxidant activity was calculated based on a standard Trolox curve 0–250 μM (r^2^ = 0.9943) [58]. All measurements were performed in fivefold.

#### 4.9.3. ORAC

To determine antioxidant activity, 80 μL of sample, 80 μL of fluorescein (14.66 ppm) and 40 μL of AAPH (120 mg/mL) were mixed in 96-well microplates. The mixture was left incubated at 37 °C in the Multilabel Plate Counter Victor^®^ 1420 (PerkinElmer, Waltham, MA, USA) for 180 min, reading fluorescence every 5 min at λex = 485 nm and λem = 535 nm. Antioxidant activity was determined using a standard Trolox 20 μM, according to Equation (6) [59]. All measurements were carried out at least five times.

(6) $$ORAC\;({\mu M}_{Trolox})=20\times K\times \frac{\left(S_{sample}-S_{blanck}\right)}{\left(S_{Trolox}-S_{blanck}\right)}$$

where *K* is the dilution factor and

(7) $$S=\left(0.5+\frac{F_{5}}{F_{0}}+\frac{F_{10}}{F_{0}}+\frac{F_{15}}{F_{0}}+\cdots \frac{F_{180}}{F_{0}}\right)\times 5$$

### 4.10. Antiproliferative Effect

#### 4.10.1. Cell Lines and Culture Conditions

Human colon cancer Caco-2 cells (HTB-37) were obtained from the American Type Culture Collection (Rockville, MD, USA) and used at passages 8–14. Cells were cultured in 75 cm^2^ flasks (Corning™ Falcon™) with DMEM medium containing 4.5 g/L glucose and supplemented with 10% *v*/*v* fetal bovine serum, 1% (*v*/*v*) nonessential amino acids, 1% (*v*/*v*) HEPES, 1% (*v*/*v*) penicillin/streptomycin and 0.1% (*v*/*v*) fungizone at a final pH of 7.2–7.4. Cells were maintained at 37 °C in an incubator under a 5% CO_2_/95% relative humidity [60]. For the tumoral model, cells were seeded and allowed to adhere for 24 h, and for the non-tumoral model, cells were seeded, and every 48 h, DMEM medium was changed until day 10, with cells in a differentiated state (indicated by a transepithelial electrical resistance (800 Ω × cm^2^) [61], when the cells were treated. All samples were diluted with DMEM (1:15, *v*/*v*) (this concentration was chosen from the concentrations 1:10, 1:20, 1:30 and 1:50 previously tested to avoid any cytotoxicity effect by the digestion process) and filtered to sterilize (0.45 μm, PTFE). Parallel cultures of untreated cells were maintained under the same conditions, and 25 µM of 5-fluorouracil (5-FU) was used as a positive control.

#### 4.10.2. MTT Cytotoxicity Assay

Amounts of 7.1 × 10^4^ cells/cm^2^ for the tumoral model and 4.2 × 10^4^ cells/cm^2^ for the non-tumoral model were seeded in 96-well microplates. Then, these treated with 200 µL of each sample diluted with D-MEM (1:15, *v*/*v*) for 24 h in all experiments. After the incubation period, the MTT cytotoxicity assay was performed to assess the metabolic activity of the cells. This assay is based on the conversion of yellow MTT tetrazolium salt to purple formazan crystals in metabolically active cells. Cells were incubated with 100 μL of MTT solution for 4 h at 37 °C, 95% RH and 5% CO_2_. The formazan crystals were then solubilized in DMSO, and absorbance was measured using a Synergy H1 microplate reader (BioTek, Agilent, Santa Clara, CA, USA) at 570 nm, with background subtraction at 690 nm. Absorbance values were correlated with cell viability and expressed as a percentage relative to the control cells. Results were expressed as % of viability compared to cell control. All measurements were carried out at least five times [60].

#### 4.10.3. Apoptosis: Annexin V/IP Assay

In 24-well plates, 5.2 × 10^4^ cells/cm^2^ were seeded and treated with 1000 µL of each sample diluted with D-MEM (1:15, *v*/*v*) for 24 h. Apoptosis was detected using a two-dimensional gating method with an FITC-Annexin V kit. This kit uses annexin V conjugated with fluorescein isothiocyanate (FITC) to bind to phosphatidylserine residues on the membrane surface, marking early apoptotic events. Propidium iodide (PI) was used to stain the DNA of necrotic cells where the cell membrane had been completely compromised. This combination enabled the differentiation of viable cells (Annexin V and PI negative), early apoptotic cells (Annexin V positive), late apoptotic cells (PI positive) and necrotic cells (Annexin V and PI positive). Cell analysis was performed using flow cytometry (FACS-Verse^Tm^, BD Biosciences, Piscataway, NJ, USA) with at least 10,000 events analyzed per sample. Measurements were conducted in triplicate over two separate days [60].

#### 4.10.4. Cell Cycle Progression

In 24-well plates, 5.2 × 10^4^ cells/cm^2^ were seeded and treated with 1000 µL of each sample diluted with DMEM (1:15, *v*/*v*) for 24 h. The cell-cycle distribution was analyzed by staining with propidium iodide (PI), which binds to DNA, enabling differentiation between cells with normal DNA content (G0/G1 phase) and those with doubled DNA content (G2/M phase). Cells in the S phase, with intermediate DNA content, were also identified, while apoptotic cells, with reduced DNA content, were categorized in the sub-G1 phase. Cells were incubated with ethanol/PBS 70:30 (*v*/*v*), followed by incubation with a mixture of RNase A (40 µg/mL) and PI (100 µg/mL) in PBS for 30 min at 37 °C, 95% RH and 5% CO_2_. Flow cytometry analysis was performed using flow cytometry (FACS-Verse^Tm^, BD Biosciences, Piscataway, NJ, USA), with at least 10,000 events analyzed per sample. Measurements were conducted in triplicate over two independent days [60].

#### 4.10.5. Levels of Reactive Oxygen Species (ROS)

In 24-well plates, 5.2 × 10^4^ cells/cm^2^ were seeded and treated with 1000 μL of every sample diluted with D-MEM (1:15, *v*/*v*) for 24 h. ROS content was measured using dichlorofluorescein diacetate (DCFDA), which is oxidized by ROS to produce fluorescence. Cells were incubated with DCFDA at a final concentration of 1 µM in the dark for 30 min at 37 °C in a 5% CO_2_ atmosphere with 95% relative humidity. After incubation, the cells were centrifuged at 450× *g* for 5 min at 25 °C, resuspended in 300 µL of PBS and analyzed by flow cytometry (FACS-Verse^Tm^, BD Biosciences, Piscataway, NJ, USA). A minimum of 10,000 events were analyzed per sample, and measurements were performed in triplicate [60].

#### 4.10.6. Intracellular Reduced Glutathione (GSH)

In 24-well plates, 5.2 × 10^4^ cells/cm^2^ were seeded and treated with 1000 μL of every sample diluted with D-MEM (1:15, *v*/*v*) for 24 h. The content of GSH was measured using 5-Chloromethylfluorescein diacetate (CMFDA), a specific dye that binds to nonprotein thiols such as GSH. A 500 µL aliquot of cell suspension was mixed with CMFDA to achieve a final concentration of 1 µM. The cells were then incubated for 40 min at 37 °C in a 5% CO_2_ atmosphere with 95% relative humidity, followed by centrifugation at 450× *g* for 5 min at 25 °C. The cells were suspended in 300 µL of PBS and analyzed by flow cytometry (FACS-Verse^Tm^, BD Biosciences, Piscataway, NJ, USA). At least 10,000 events were analyzed for each sample for each sample and measurements were performed in triplicate.

### 4.11. Statistics

The results are presented as mean ± standard deviation (SD). Statistical comparisons of the means were performed using ANOVA, followed by Fisher’s least significant difference (LSD) test, with a 95% confidence level. The Simplex lattice mixture design was applied for data analysis using STATGRAPHICS 19^®^ (The Plains, VA, USA).

## Figures and Tables

**Table 1:** Results for the mixture experimental design.

Run	Polyphenol Bioaccessibility (%)	Carotenoid Bioaccessibility (%)	Polyphenol EE (%)	Particle Size (µm)	ABTS (%)	FRAP (%)	Sphericity
1	105.16	75.79	64.7	1539.27	123.01	57.83	0.71
2	126.59	50.96	40.8	2224.47	44.02	72.7	0.66
3	110.20	61.34	49.4	2078.63	47.59	97.39	0.63
4	132.54	88.24	46.2	1625.8	43.78	51.06	0.66
5	156.93	105.41	51.4	1825.81	78.14	90.1	0.67
6	85.37	70.11	57.1	1866.22	129.55	91.24	0.76
7	73.48	85.42	58.5	1701.86	71.28	168.48	0.68
8	142.13	97.26	59.8	1592.14	34.41	106.78	0.66
9	135.70	55.38	49.6	1349.00	93.86	120.17	0.72
10	125.98	59.81	51.1	1149.88	95.02	130.33	0.65
11	77.55	62.50	55.9	1787.01	127.7	49.99	0.74
12	109.97	74.70	52.8	1720.6	55.79	170.53	0.69
*p*-value	0.0341	0.1823	0.0274	0.0313	0.0111	0.0374	0.0032
r^2^	90.74	92.27	82.74	95.48	91.27	85.42	94.77
r^2^-adjust	79.18	72.93	68.35	87.95	82.54	70.84	89.54

**Table 2:** Antioxidant activity for the fruit blend (F), fruit blend hydrogel bead (FHB) and hydrogel beads (HBs).

	F	FHB	HB
Total Polyphenols	µg GAE/100 g		
Non-digested	23,807.69 ± 783.29 ^a^	11,404.89 ± 335.04 ^c^	153.21 ± 43.33 ^d^
Bioaccessible fraction	26,946.15 ± 1201.58 ^b^	25,346.15 ± 1786.21 ^a, b^	16,330.77 ± 554.70 ^e^
ABTS	µM Trolox/100 g		
Non-digested	91.50 ± 1.43 ^a^	69.33 ± 7.11 ^b^	16.81 ± 3.56 ^d^
Bioaccessible fraction	75.85 ± 16.293 ^b^	47.41 ± 0.00 ^c^	15.41 ± 2.51 ^d^
FRAP	µM Trolox/100 g		
Non-digested	77.34 ± 1.31 ^a^	22.13 ± 1.92 ^c^	7.23 ± 3.18 ^d^
Bioaccessible fraction	72.85 ± 2.60 ^b^	20.54 ± 2.40 ^c^	6.70 ± 0.71 ^d^
ORAC	µM Trolox/100 g		
Non-digested	47.06 ± 9.31 ^a^	32.10 ± 4.73 ^c^	7.32 ± 1.79 ^e^
Bioaccessible fraction	7865.90 ± 1238.55 ^b^	15,382.15 ± 2781.93 ^d^	7362.72 ± 2692.69 ^b^

For every test, different letters indicate statistically significant differences (*p* < 0.05). GAE: Gallic acid equivalent.

**Table 3:** Polyphenols identified in extracts of goldenberry and purple passion fruit.

Compound	Molecular Formula	Molecular Weight (*m*/*z*)	Compound	Molecular Formula	Molecular Weight (*m*/*z*)
Apigenin	C_15_H_10_O_5_	271.06010	kaempferol 3-glucoside	C_21_H_20_O_11_	449.10784
Artepillin C	C_19_H_24_O_3_	301.17982	Luteolin	C_15_H_10_O_6_	287.05501
Caffeic acid	C_9_H_8_O_4_	181.04954	Narigenin	C_15_H_12_O_5_	273.07575
Caffein	C_8_H1_0_N_4_O_2_	195.08765	Orientin	C_21_H_20_O_11_	449.10784
Carnosic acid	C_20_H_28_O_4_	333.20604	p-coumaric acid	C_9_H_8_O_3_	165.05462
Catechin	C_15_H_14_O_6_	291.08631	Pelargonidin	C_15_H_11_O_5_^+^	271.0601
Chlorogenic acid	C_16_H_18_O_9_	355.10236	pelargonidin 3-glucoside	C_21_H_21_O_10_^+^	433.11292
Cinnamic acid	C_9_H_8_O_2_	149.05971	Pinocembrin	C_15_H_12_O_4_	257.08084
Cyanidin	C_15_H_11_O_6_^+^	287.05501	Protocatechuic acid	C_7_H_6_O_4_	155.03389
Cyanidin 3-rutinoside	C_27_H_31_ClO_15_	631.14242	Quercetin	C_15_H_10_O_7_	303.04993
Epicatechin	C_15_H_14_O_6_	291.08631	Quercetin-3-glucoside	C_21_H_20_O_12_	465.10275
Epigallocatechin	C_15_H_14_O_7_	324.10778	Rosmarinic acid	C_18_H_16_O_8_	361.09179
Eriodictyol	C_15_H_12_O_6_	289.0706	Rutin	C_18_H_16_O_8_	611.16066
Ferulic acid	C_10_H_10_O_4_	195.06519	Theobromine	C_7_H_8_N_4_O_2_	181.0720
Gallic acid	C_7_H_6_O_5_	171.0288	ursolic acid	C_30_H_48_O_3_	457.36762
Isoorientin	C_21_H_20_O_11_	449.10784	Vanilin	C_8_H_8_O_3_	153.05462
Isovitexin	C_21_H_20_O_10_	433.11292	Vanillic acid	C_8_H_8_O_4_	169.04954
Kaempferol	C_15_H_10_O_6_	287.05501			

**Table 4:** Simplex lattice mixture design for the formulation of hydrogel beads.

Run	SA	HPMC	AG	Run	SA	HPMC	AG
1	0.50	0.5	0.00	7	0.75	0.25	0.00
2	0.50	0.00	0.50	8	0.75	0.00	0.25
3	0.50	0.25	0.25	9	0.58	0.33	0.08
4	0.58	0.08	0.33	10	0.67	0.17	0.17
5	0.83	0.08	0.08	11	1.00	0.00	0.00
6	1.00	0.00	0.00	12	0.75	0.25	0.00

SA: sodium alginate; HPMC: hydroxypropyl methylcellulose; AG: Arabic gum.

## Data Availability

The data presented in this study are available upon reasonable request from the corresponding author.
